# Feasibility and efficacy of spinal microtubular technique for resection of lumbar dumbbell-shaped tumors

**DOI:** 10.3389/fonc.2022.1024877

**Published:** 2022-11-07

**Authors:** Rui Wang, Zeyan Liang, Yan Chen, Xiongjie Xu, Chunmei Chen

**Affiliations:** Department of Neurosurgery, Fujian Medical University Union Hospital, Fuzhou, China

**Keywords:** lumbar dumbbell-shaped tumor, microtubular technique, paravertebral approach, minimally invasive surgery, Eden classification

## Abstract

**Objective:**

Minimally invasive surgical resection of lumbar dumbbell-shaped tumors is rarely reported. We retrospectively collected clinical data of lumbar dumbbell-shaped tumors treated with the spinal microtubular technique to evaluate the feasibility, complications and efficacy of the surgical methods.

**Methods:**

From September 2013 to August 2021, clinical data of patients with lumbar dumbbell-shaped tumors that underwent paravertebral approach and micro-tubular tumorectomy (PAMT) were collected; neurological function was assessed using the pain visual analog scale (VAS) and the Japanese Orthopaedic Association (JOA) score.

**Results:**

A total of 46 patients that underwent PAMT were included in this study. In all patients, total resection of the tumor was performed at one stage (100%). The median follow-up period was 27.5 months (P25, P75: 16.5- 57 months). Symptoms such as pain or lower extremity weakness were significantly relieved in 46 patients. The postoperative VAS score and JOA score were significantly higher compared with preoperative scores (*p <*0.001), and the patients had no tumor recurrence or spinal instability. According to the Eden classification, there were 7 cases of type I, 8 cases of type II, 15 cases of type III, and 16 cases of type IV. In the comparison of the improvement of VAS score at 12 months after PAMT, there were significant differences among different types of tumors (H =15.756, *p* =0.001); type I was better than type III (Z =2.768, *p* =0.029) and type IV (Z =2.763, *p* =0.029), and type II was also better than type III (Z =2.679, *p* =0.037) and type IV (Z =2.708, *p* =0.034). With respect to estimated blood loss (Z =-3.041, *p* =0.013) and postoperative hospital stays (Z =-3.003, *p* =0.014), type IV was less than type III; and type IV was also less than type II about operation time (Z =-2.653, *p* =0.040).

**Conclusion:**

In small lumbar dumbbell-shaped tumors, PAMT is indicated for Eden types I-IV and different pathological types of tumors, and can achieve complete resection of the tumor (GTR) in one stage with a good prognosis.

## 1 Introduction

Among spinal tumors, lumbar dumbbell-shaped tumors are relatively rare, and the preferred treatment is surgical resection of the tumor. In the treatment of lumbar dumbbell-shaped tumors, total resection of the tumor and preservation of spinal stability are the main concerns of the surgeon. Surgeons choose more traditional surgeries and rarely choose minimally invasive surgery, in order to balance total resection of the tumor and preservation of spinal stability. Traditional surgery often requires resection of the facet joint to adequately expose and completely remove the tumor, which results in the need to apply instrumentation to preserve spinal stability. Intraoperative instrumentation leads to additional tissue damage, prolonging the patient’s recovery time after surgery and potentially adding new symptoms. In the treatment of lumbar dumbbell-shaped tumors, we used the paravertebral approach and micro-tubular tumorectomy (PAMT) for one-stage tumor resection. The Eden classification and tumor pathological type of lumbar dumbbell-shaped tumors are decisive factors in determining surgical options. The Eden classification method is currently one of the most common methods for the clinical classification of spinal dumbbell-shaped tumors. Based on the combination of MRI and CT findings, the EDEN classification is more accurate in identifying the type of thoracolumbar dumbbell tumors and helps surgeons to select a better surgical approach (1, 2). Most patients with lumbar dumbbell-shaped tumors had a history of low back pain or leg pain, and improvement of symptoms after surgery is one of the main objectives of patients and surgeons. the VAS score and the JOA score were often used as indicators to assess the degree of pain symptoms and recovery in spine patients, so we chose these two scoring systems to evaluate and analyze the improvement of patients’ symptoms before and after surgery. We collected clinical data on 46 patients with lumbar dumbbell-shaped tumors over the last 8 years. In this group of cases, slightly different tubular spinal approaches were selected by applying Eden classification to achieve total resection of the tumor while preserving spinal stability. The feasibility and efficacy of the procedure were evaluated by analyzing the perioperative data, postoperative neurological function, and complications. On the other hand, the limitations of the minimally invasive tubular spinal approach were discussed to provide a reference for surgeons in selecting treatment options.

## 2 Materials and methods

### 2.1 Patients

From September 2013 to August 2021, 46 cases of lumbar dumbbell-shaped tumors were collected from the department of neurosurgery, Fujian medical university union hospital. Each patient underwent lumbar X-ray hyperextension and flexion, lumbar CT three-dimensional reconstruction, and lumbar magnetic resonance imaging (MRI) before and after surgery. The inclusion criteria for this study were as follows: (1) The tumor was Eden type I-IV; (2) The tumor diameter in the spinal canal was ≤2cm; (3) The diameter of the paravertebral tumor was ≤5cm; (4) Signed the patient authorization form. The exclusion criteria were as follows: (1) The tumor invaded ≥2 intervertebral foramen; (2) The length of the tumor tissue in the spinal canal was more than 2 segments or the tumor invaded the spinal cord; (3) history of surgery in the segment where the tumor was located; (4) vascular tumor or tumor with abundant blood flow; (5) Lumbar instability or scoliosis deformity. Approval for this study was granted by the Ethics committee of Fujian Medical University Union Hospital, Fuzhou, China (2020YF023-01).

### 2.2 Intraoperative neurophysiological monitoring method and early warning criteria

Intraoperative neurophysiological monitoring was selected based on the patient’s voluntary principle. The EpochXP (system, USA) neurophysiological monitoring system was used intraoperatively. The spinal somatosensory evoked potential (SSEP), transcranial motor evoked potential (TcMEP) and spontaneous electromyography (EMG) were continuously monitored intraoperatively with the healthy side as the control. SSEP and TcMEP were used to monitor the lower limb and anal sphincter; EMG was performed at the appropriate level of spinal nerve innervation. The electrode placement and stimulation parameters were selected according to the lesion segment and the surgical procedure (3). SSEP and TcMEP were defined as a prolongation of latency ≥ 10% or a decrease in amplitude ≥ 50%; EMG was defined as a burst of EMG activity or continuous EMG activity.

### 2.3 Surgical procedure

#### 2.3.1 Anesthesia and position

After the induction of general anesthesia, the patients were placed in a prone position to minimize lumbar lordosis/thoracic kyphosis and avoid compressing the abdomen.

#### 2.3.2 Approach and exposure

Pre-operative design of the incision, location, and direction of the micro-tubule based on MRI and other imaging data.

**Figure 1:**
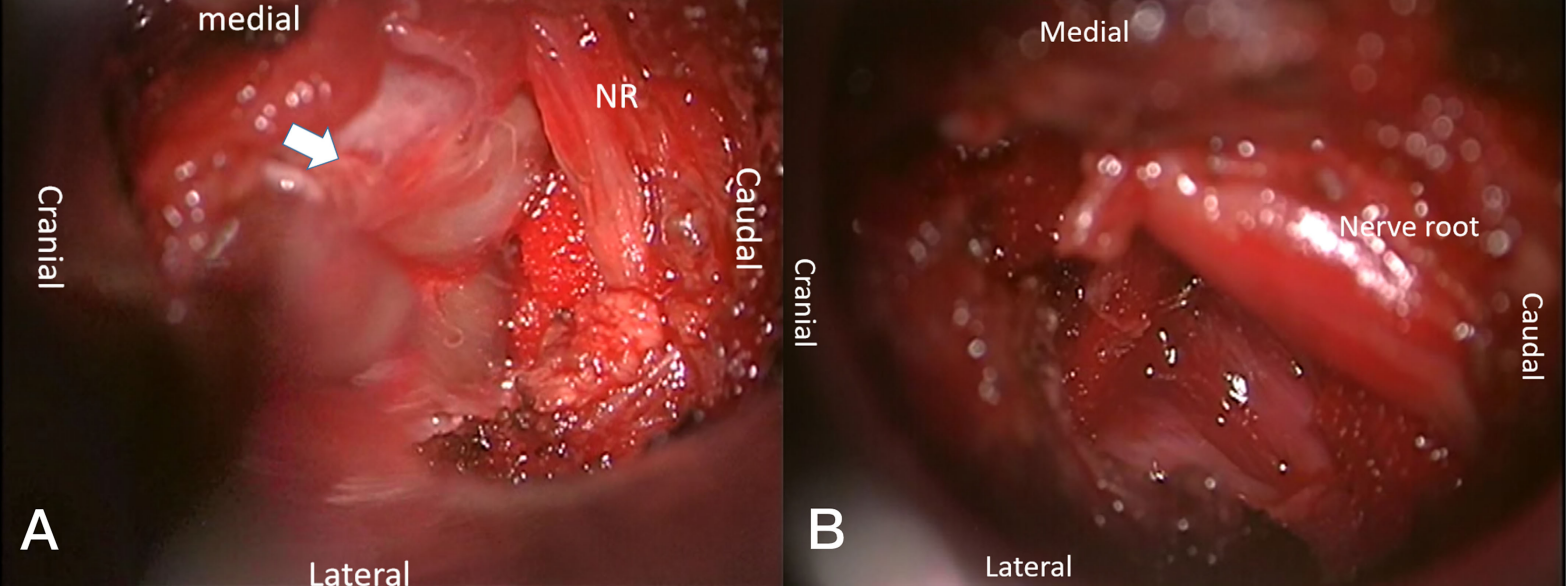
Tumor resection of Eden 4 type by PAMT. After inserting the micro-tubule, the relevant anatomy was exposed **(A)**: paravertebral tumor (white arrow) and nerve root (NR). The nerves remained intact after tumor resection **(B)**.

A 20–25 mm skin incision was made approximately 20–35 mm lateral to the midline (adjusted according to the patient’s body habitus), and the accurate target level was confirmed by X-ray fluoroscopy. The paravertebral approach involved the paravertebral muscles, which were bluntly dissected using the muscle splitting technique. After the smallest dilator was inserted to reach the lamina or peripheral bone structure, the dilators were sequentially placed on top of each other, and a working tubule (diameter, 14 mm or 16 mm) was inserted over the dilators. The dilators were removed, and a tubular surgical path was established. The tubule was fixed using a flexible arm mounted on the operating table. Because of the flexible fixed arm, the tubule can be angulated to expand the operating field (**Figure 1**). The subsequent procedure was performed under a microscope (OPMI Pentero; Carl Zeiss, Germany).

#### 2.3.3 Typical steps

**Figure 2:**
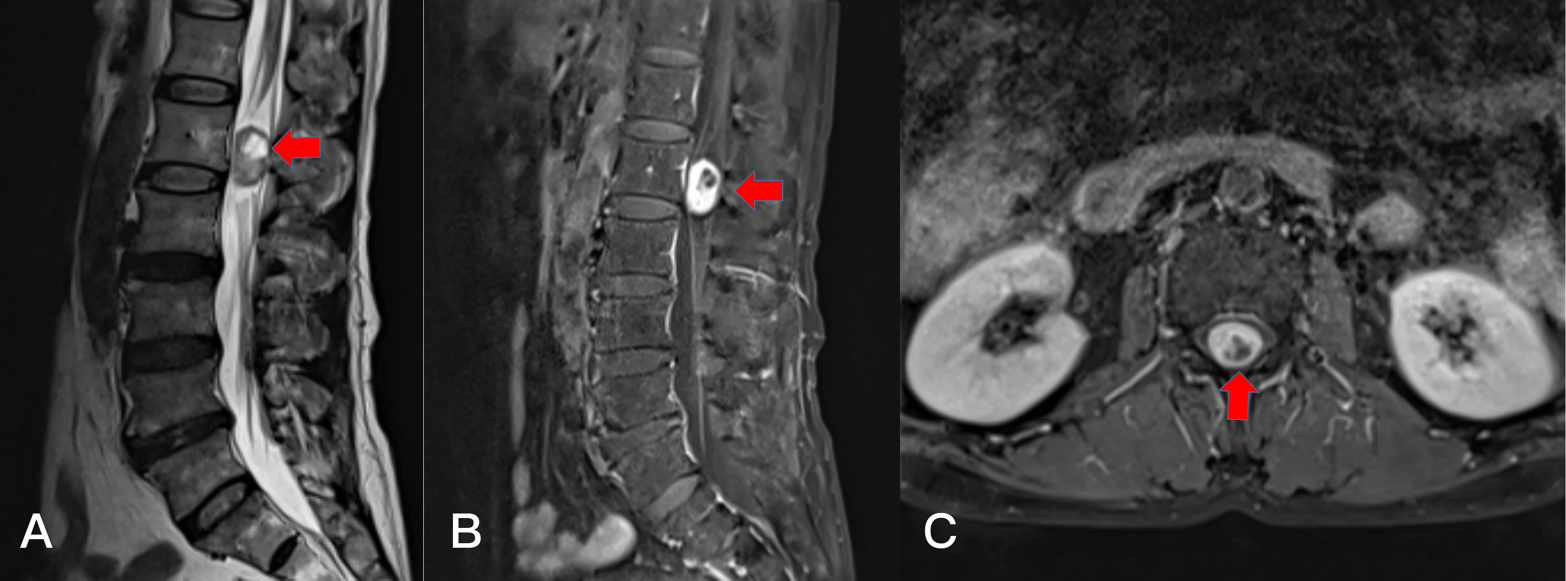
Preoperative T2-weighted MRI on sagittal **(A)** plane, T1-weighted enhanced sagittal **(B)** plane, and axial **(C)** planes, revealing a subdural tumor in the L1-2 segment (red arrows).

**Figure 3:**
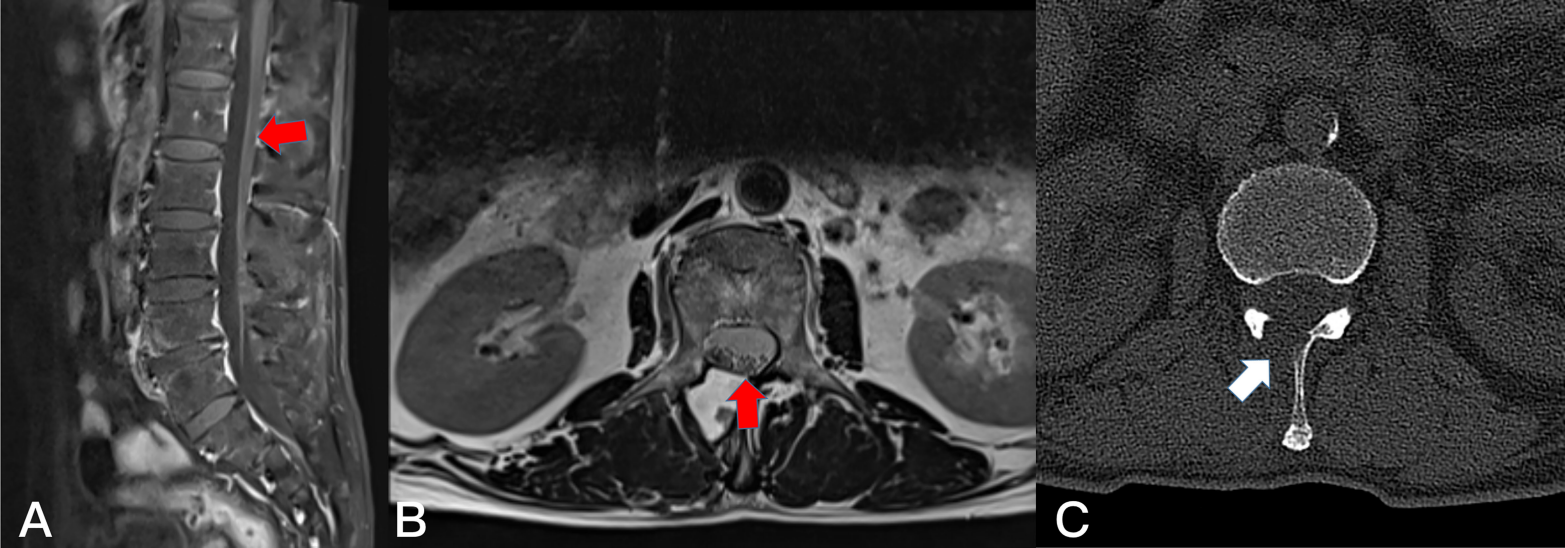
Postoperative T1-weighted enhanced MRI on sagittal **(A)** planes and T2-weighted MRI on axial **(B)**. Resection of the subdudral tumor could not be identified after 1 months (red arrows). The axial plane **(C)** of CT scan showed the bone window of L1 lamina (white arrows).

In Eden I tumors, the microtubules reached the lamina to facilitate intraspinal tumor removal. A high-speed drill combined with Kerrison punches was used to remove part of the lamina, and the ligamentum flavum was excised to expose the dura and the spinal nerves. The dura and nerve sleeve were incised in an “L” shape, and the tumor tissue was excised after cutting the tumor-bearing nerve root and tumor blood vessels to protect the normal nerve root. If the tumor was large, the tumor can be resected in pieces (**Figures 2**, **3**).

**Figure 4:**
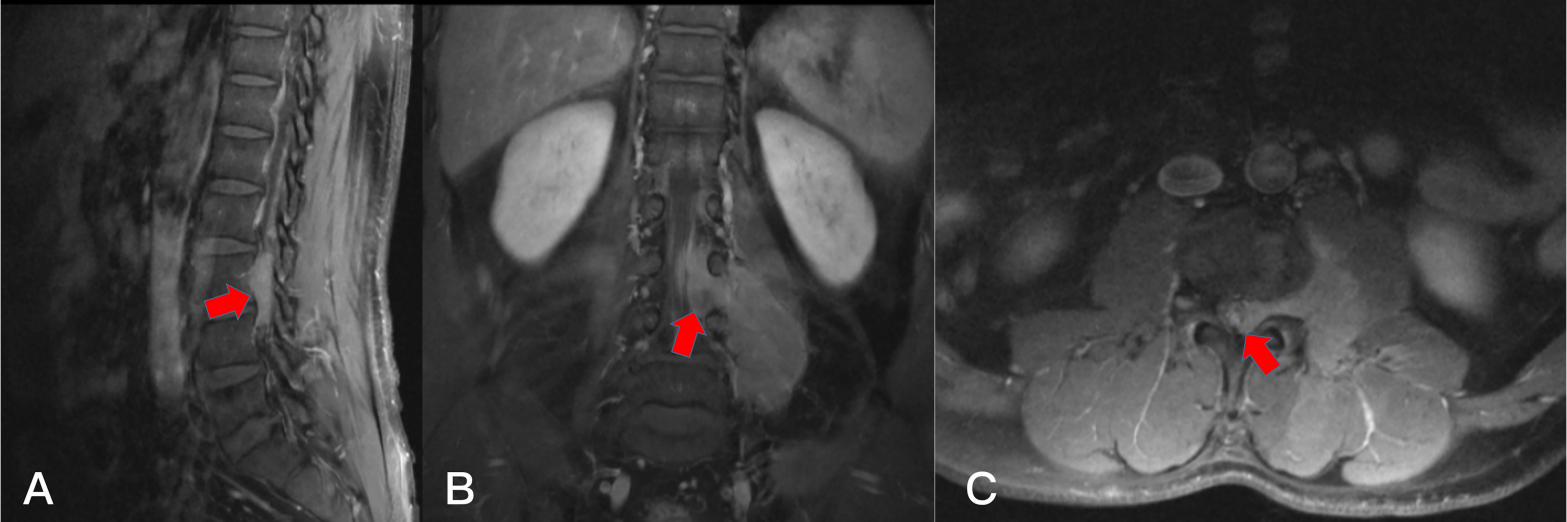
Preoperative T1-weighted enhanced MRI on sagittal **(A)** plane, coronal **(B)** plane, and axial **(C)** plane, revealing a dumbbell tumor (Eden II) in the left L3-4 foramen (red arrows).

**Figure 5:**
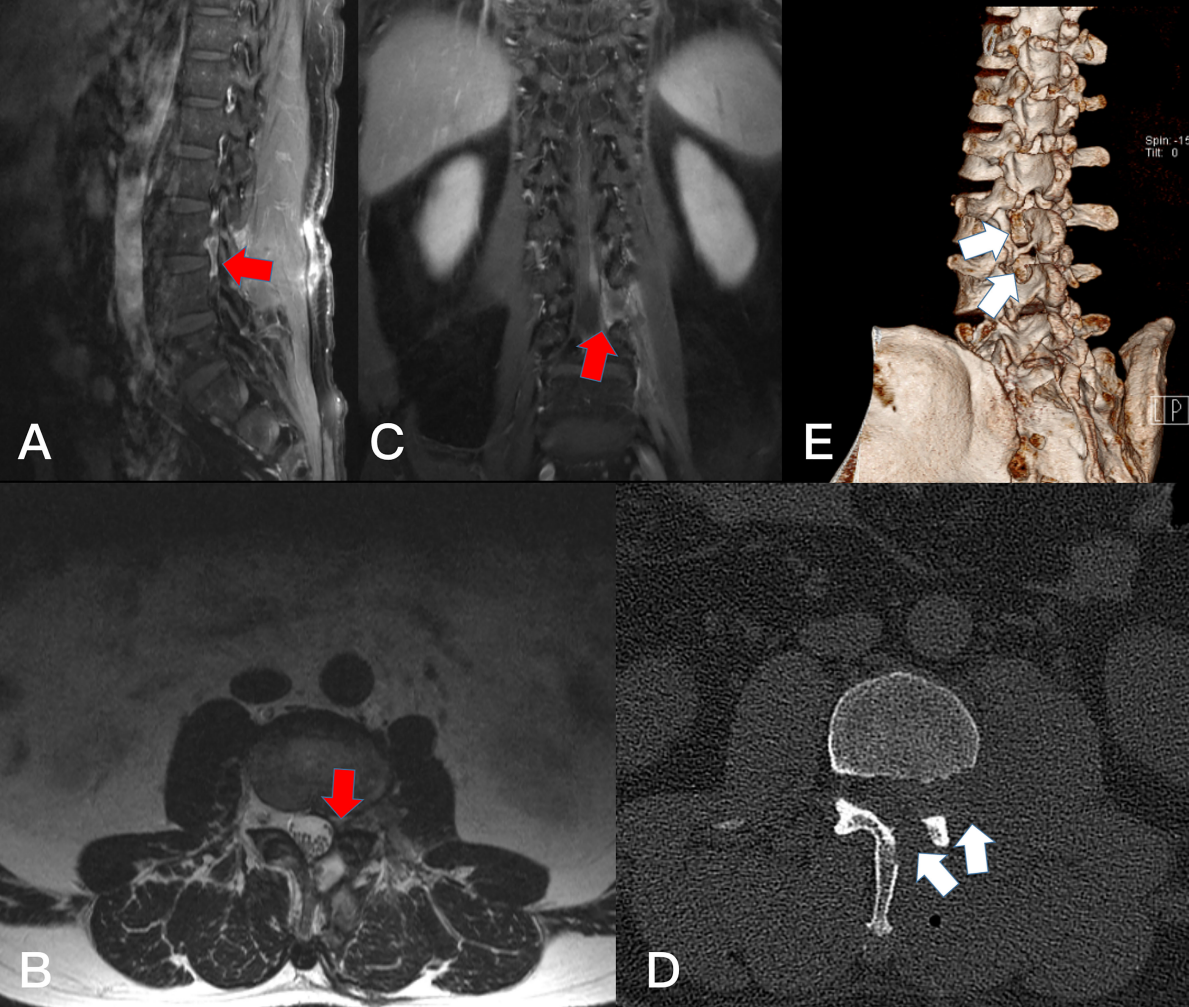
Postoperative T2-weighted MRI on sagittal **(A)**, axial**(B)**, and T1-weighted enhanced on coronal **(C)** plane, Resection of the dumbbell tumor could not be identifiedafter 6 months (red arrows). The axial plane and 3D-CT scan showed the bone window of L3 and L4 lamina **(D, E)** (white arrows).

In Eden II and Eden III tumors, tumor resection was first performed in the intraspinal tumor, followed by the paraspinal tumor. Excision of part of the lamina and ligamentum flavum to expose the dura and nerve roots was performed as described above. In the case of Eden II, the dura mater and nerve sleeve were incised in an “L” shape, the subdural tumor tissue was excised in pieces, the dura was closed with 6-0 nylon sutures, and the processing steps of resecting epidural tumor tissue and paravertebral tumor tissue were the same as Eden III (**Figures 4**, **5**).

**Figure 6:**
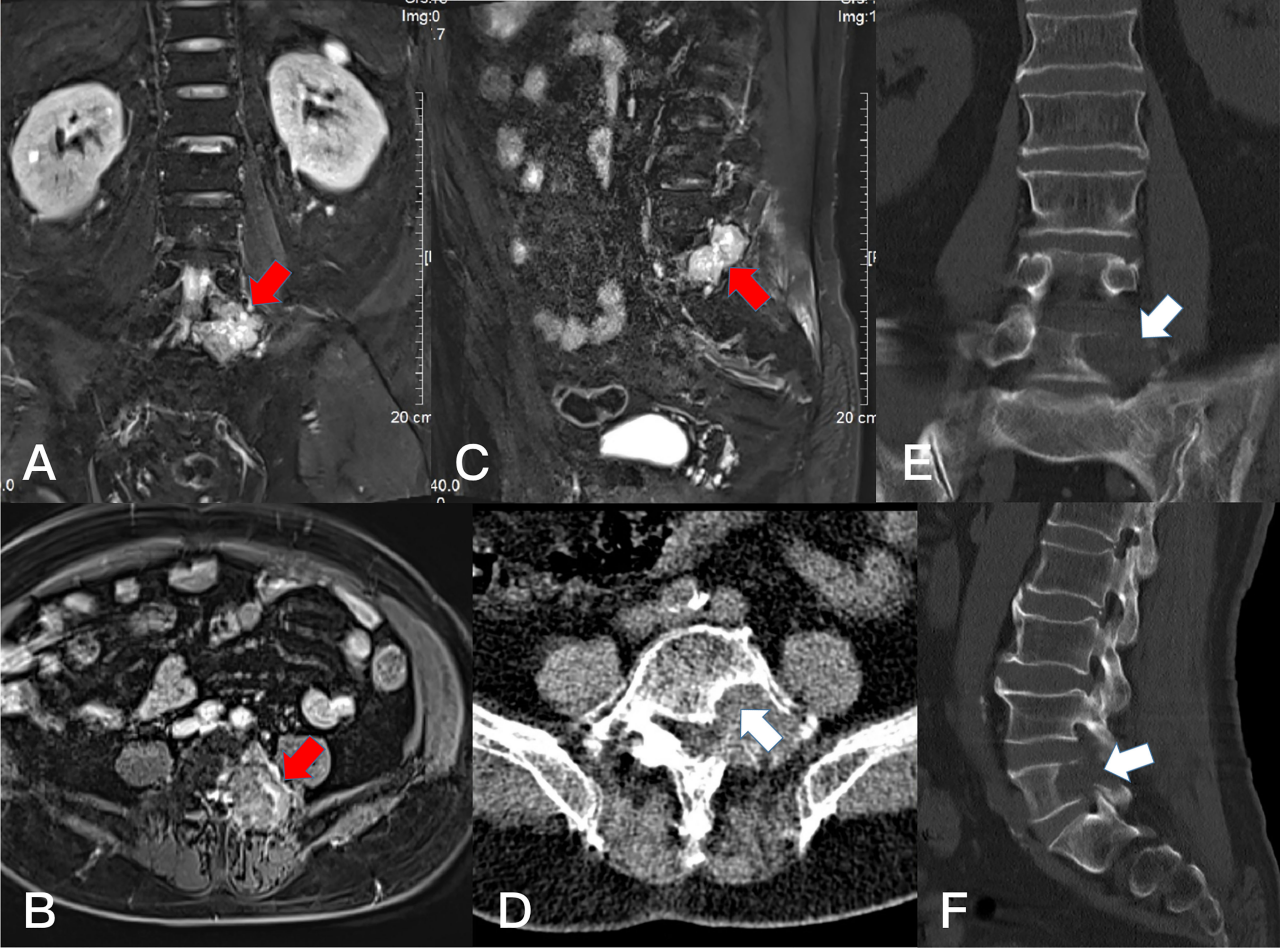
Preoperative T1-weighted enhanced MRI on coronal **(A)** plane, axial **(B)** plane, and sagittal **(C)** plane, revealing a dumbbell tumor (Eden III) in the left L5-S1 foramen (red arrows). The CT scan on axial **(D)** plane, coronal **(E)** plane, and sagittal **(F)** plane showed the bone damage of left lamina, facet joint and vertebral body of L5(white arrows).

**Figure 7:**
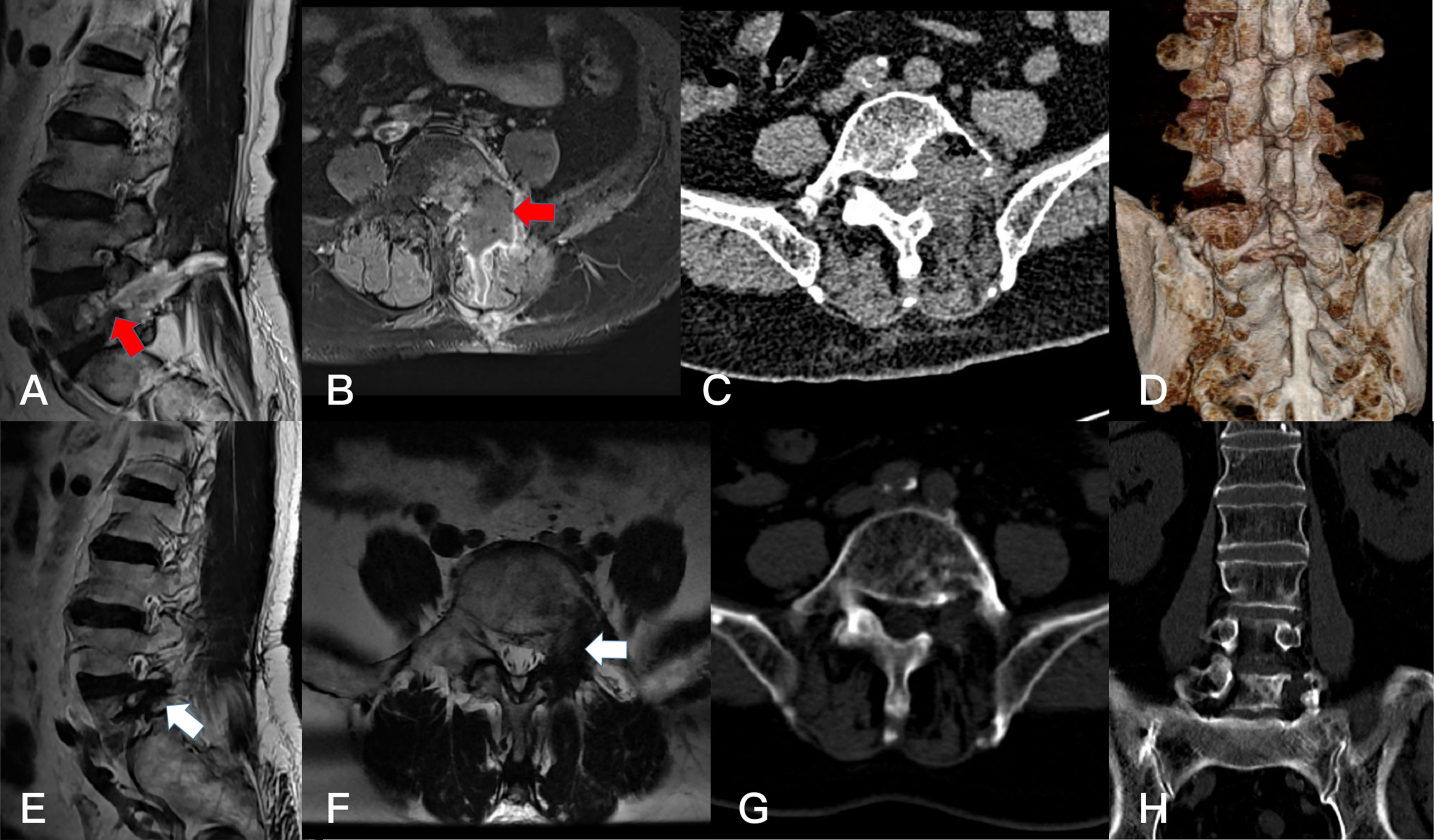
Postoperative T1-weighted enhanced MRI on sagittal **(A)** plane, and axial **(B)** palne, and the axial **(C)** and 3D-CT **(D)** scan. Resection of the dumbbell acute lymphocytic leukoma (B cell) could not be identified after 1 months (arrows). Postoperative T1-weighted enhanced MRI on sagittal **(E)** plane, and axial **(F)** palne, the CT scan of the axial **(G)** plane and coronal **(H)** plane after 2 years, showing the resection of the dumbbell tumor could not be identified (arrows).

**Figure 8:**
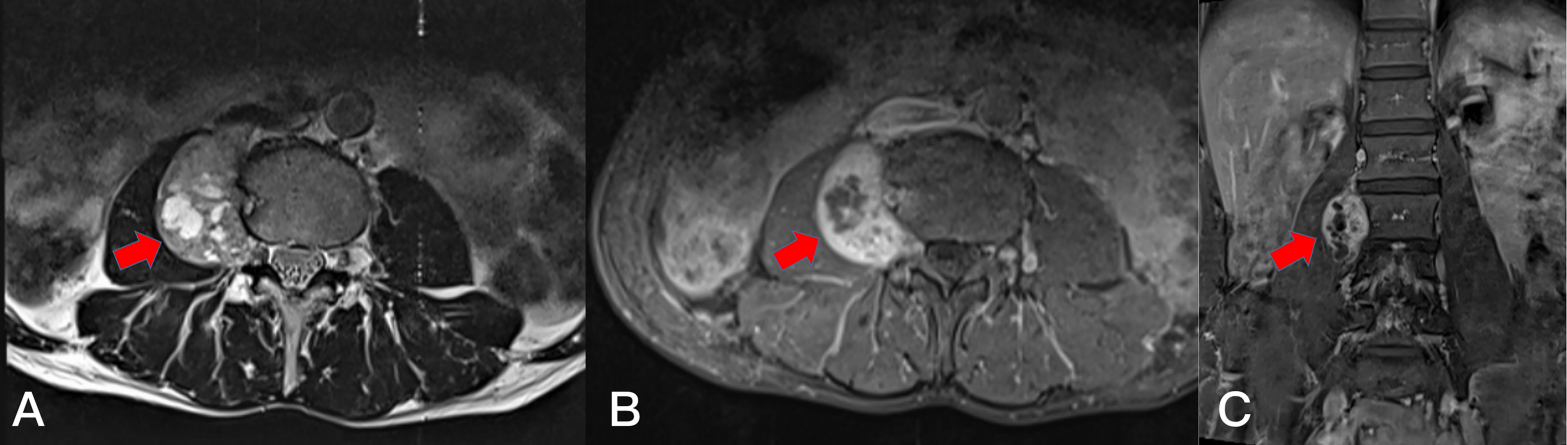
Preoperative T2-weighted MRI on axial **(A)** plane, T1-weighted enhanced MRI on axial **(B)** plane and sagittal **(C)** plane, revealing a dumbbell tumor (Eden IV) in the right L3-4 segment (red arrows).

**Figure 9:**
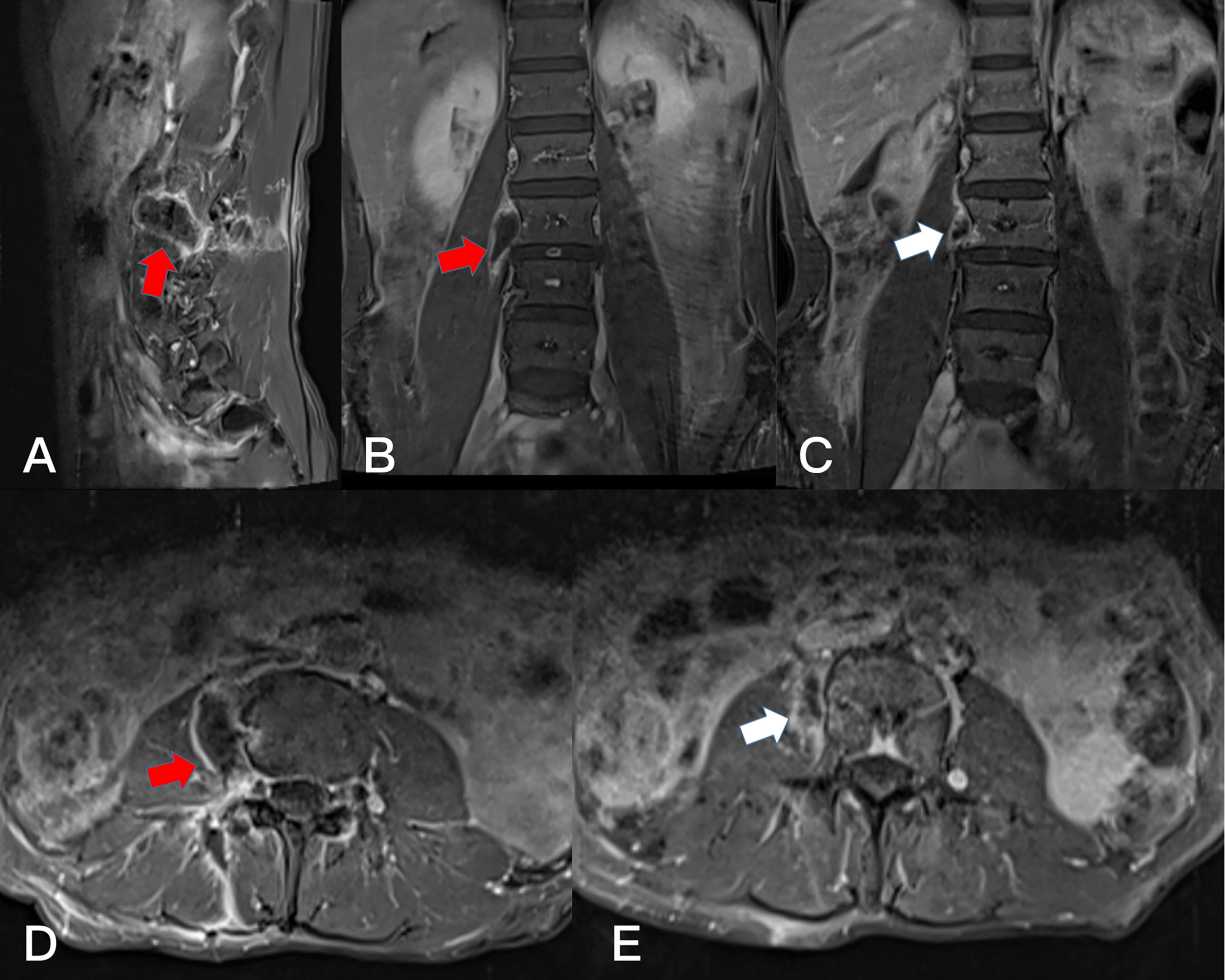
Postoperative T1-weighted enhanced MRI on sagittal **(A)**, coronal **(B)**, and axial **(D)** plane. Resection of the dumbbell tumor could not be identified after 1 month (arrows). Postoperative T1-weighted enhanced MRI on coronal **(C)**, and axial **(E)** plane. Resection of the dumbbell tumor could not be identified after 1 year (arrows).

Eden III tumors were located in the epidural space, the intraspinal and foraminal tumors were resected in pieces, and the tumor-bearing nerve with obvious tumorization was removed. The bone window of the spinal canal was temporarily sealed using a gelatin sponge. If the diameter of the paravertebral tumor is ≤2 cm, the direction of the tubule can be directly adjusted to expose and remove the paravertebral tumor. If the diameter of the paravertebral tumor was ≥2 cm, the microtubule must be reinserted to reach the region between transverse processes and establish a second paravertebral muscle tubular path (dual-tubule path); if necessary, remove the part of the transverse process and expose and remove the extra-foraminal tumor and paravertebral tumor. (**Figures 6**, **7**) For Eden IV tumors, the microtubule directly reaches the paravertebral transverse process, exposing and resecting the extraforaminal tumor and paravertebral tumor. The paravertebral muscles were repositioned, and the muscle fascia, subcutaneous tissue, and skin were sutured layer-by-layer. (**Figures 8**, **9**)

*Postoperative Measurements*

The drainage tube was removed within 24 h after surgery, and patients underwent passive lower extremity training and walking training protected by a belt brace as soon as possible. A belt brace was used for 1–2 weeks after surgery.

### 2.4 Outcome measurements and data collection

Neurological improvement was assessed using the pain visual analog scale (VAS) and Japanese Orthopaedic Association (JOA) scores. The baseline data included sex, age, body mass index (BMI), comorbidities, target segment, clinical performance, and preoperative VAS and JOA scores. The primary outcome measure was the Gross total resection (GTR) rate. The extent of resection was defined as GTR if there was no residual tumor on postoperative MR images and Near-total resection (NTR) if the residual tumor was present.11 Secondary outcome indicators included operation time, estimated blood loss (EBL), postoperative hospital stays, postoperative VAS score, postoperative JOA score, tumor recurrence rate, and spinal stability during the follow-up period.

### 2.5 Statistical analysis

Data were analyzed using JMP^®^ Trial 16.2.0 (SAS software). Frequencies were used to describe the general data and disease investigation status of patients with lumbar dumbbell-shaped tumors; medians were used to describe continuous variables. The Kruskal–Wallis non-parametric test was used to investigate the effect of the types of tumors in relation to the efficacy and safety of PAMT, and the Dwass-Steel-Critchlow-Fligner (DSCF) test was used for multiple comparative analyses. In addition, *p <*0.05 was considered statistically significant. *A priori* power analysis was performed using G power 3.1. The statistical test was Kruskal–Wallis non-parametric test. Therefore, we decided to use 1-sided testing, α = 0.05, and β = 0.2. According to the Cohen’s recommendation, calculate the sample size using a large effect size (0.4) (4). Then, it was determined that 42 patients were needed to detect the types of tumors in relation to the efficacy and safety of PAMT.

## 3 Results

### 3.1 General characteristics

There were 24 males and 22 females, with an median age of 49 years (P25, P75; 37, 62.8), 24 cases of nerve root symptoms, 26 cases of low back pain, 4 cases of lower extremity weakness, and 7 cases of asymptomatic. According to Eden classification (2), there were 7 cases of type I, 8 cases of type II, 15 cases of type III, and 16 cases of type IV. In terms of tumor site, there were 7 cases in L1-L2 segment, 10 cases in L2-L3 segment, 13 cases in L3-L4 segment, 12 cases in L4-L5 segment, and 4 cases in L5-S1 segment. In all 46 patients, total resection of the tumor was performed at one stage (100%). Pathological diagnoses were schwannoma (n =26), metastatic tumor (n =6), cyst (n =4), gangliocytoma (n =2), neurofibroma (n =2), granuloma (n =2), cavernous hemangioma (n =2), lipoma (n =1) and malignant neurofibroma (n =1) (**Table 1**).

**Table 1:** Clinical data of patients Measurement.

Items	Measurement
Gender, n (%)	
Male Female	24 (52.2) 22 (47.8)
Age (yrs), median (P25, P75)	49 (37, 62.8)
Eden type, n (%)	
Type I	7 (15.2)
Type II	8 (17.4)
Type III	15 (32.6)
Type IV	16 (34.8)
Tumor size (cm), median (P25, P75)	3.5 (2.5, 4.0)
Pathology, n (%)	
Schwannoma	26 (56.5)
Metastatic tumor	6 (13.0)
Cyst	4 (8.7)
Gangliocytoma	2 (4.3)
Neurofibroma	2 (4.3)
Granuloma	2 (4.3)
Angiocavernoma	2 (4.3)
Lipomyoma	1 (2.2)
Malignant neurofibroma	1 (2.2)
Tumor site, n (%)	
L1–L2	7 (15.2)
L2–L3	10 (21.7)
L3–L4	13 (28.3)
L4–L5	12 (26.1)
L5–S1	4 (8.7)
Presenting symptoms, n (%)	
Back pain	26 (56.5)
Weakness	4 (8.7)
Leg pain	24 (52.2)
asymptomatic	7 (15.2)
Surgical data, median (P25, P75)	
Operation time (min)	110 (90.0, 130.0)
EBL (ml) *	85 (60.0, 112.5)
PHS (days) *	6 (5, 7)
GTR, n (%)*	46 (100)
Complication, n (%)	
CSF leakage*	2 (4.3)
Wound infection	3 (6.5)
Cavity effusion	5 (10.9)
Spinal instability	0 (0)
Follow-up (months), median (P25, P75)	27.5 (16.5, 57)

*EBL, estimated blood loss; PHS, postoperative hospital stays; GTR, Gross total resection; CSF, Cerebrospinal Fluid.

### 3.2 Clinical improvement and follow-up

**Figure 10:**
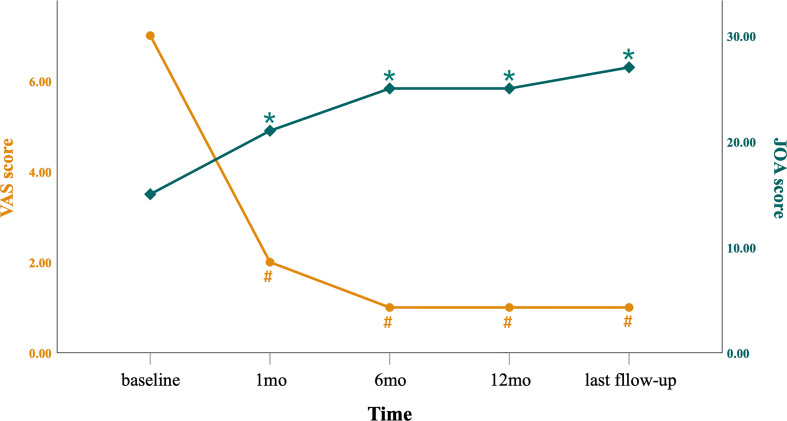
Preoperative and postoperative VAS and JOA scores. Over time, the VAS score decreased gradually and the JOA score increased gradually. #P <0.001 when comparing VAS scores with those of preoperative evaluation; *P <0.001 when comparing JOA scores with those of preoperative evaluation. mo, month.

Symptoms of 46 patients, such as pain and lower extremity weakness, were significantly relieved after surgery, and no new neurological dysfunction appeared. The median follow-up period was 27.5 months (P25, P75: 16.5, 57 months). The postoperative VAS score and JOA score were significantly higher compared with preoperative scores (*p <*0.001, see **Figure 10**), and the patients had no tumor recurrence or spinal instability. In the comparison of the improvement of VAS socre at 12 months after PAMT, there were significant differences among different types of tumors (H =15.756, *p* =0.001) (**Table 2**); type I was better than type III (Z =2.768, *p* =0.029) and type IV (Z =2.763, *p* =0.029), and type II was also better than type III (Z =2.679, *p* =0.037) and type IV (Z =2.708, *p* =0.034) (**Table 3**). According to Kruskal–Wallis non-parametric tests, there was no significant difference of JOA score among different types of tumors (*p >*0.05) (**Table 2**).

**Table 2:** Results of clinical outcomes and surgical data among patients with different Eden types.

Variables	Classification				H values	*p* values
	Type I	Type II	Type III	Type IV		
VAS score, median (P25, P75)						
Baseline	8 (7, 8)	8 (7, 8)	7 (6, 8)	7 (3, 7.75)	–	–
1 mo*	6 (4, 6)	6.5 (5.25, 7)	4 (3, 6)	2.5 (-1, 6)	6.375	0.095
6 mo*	7 (6, 7)	7 (6, 7)	6 (4, 6)	5.5 (1.25, 7)	10.313	**0.016**
12 mo*	8 (7, 8)	7 (7, 8)	6 (5, 7)	6 (2, 7)	15.756	**0.001**
Last follow-up*	7 (7, 8)	7.5 (6, 8.75)	6 (5, 7)	6 (2.25, 7)	7.099	0.069
JOA score, median (P25, P75)						
Baseline	14 (11, 17)	13.5 (11.25, 15)	15 (12, 17)	17 (14, 25)	–	–
1 mo*	6 (5, 10)	7.5 (6, 12.25)	7 (5, 8)	4 (1, 8)	3.941	0.268
6 mo*	8 (7, 13)	10.5 (9.25, 14.75)	8 (5, 11)	5.5 (1.25, 10.75)	7.274	0.064
12 mo*	10 (9, 14)	12 (7.5, 15)	8 (5, 12)	7 (2.25, 12.75)	5.212	0.157
Last follow-up*	11 (11, 15)	12.5 (11.25, 15)	10 (8, 13)	10 (2, 12.75)	6.219	0.101
Surgical data, median (P25, P75)						
Operation time (min)	110 (90,130)	125 (102.5, 187.5)	130 (100, 140)	90 (70, 107.5)	9.607	0.022
EBL (ml)	80 (50,100)	95 (82.5, 117.5)	120 (90, 180)	60 (52.5, 80)	12.386	0.006
PHS (days)	6 (5,7)	7 (6, 8.75)	6 (5, 7)	5 (4, 5.75)	10.581	0.014

* Data are presented as median values of changes in VAS scores and JOA scores from baseline at each follow-up point.

Bold values means p< 0.05.

**Table 3:** Influence of different types on VAS socre of patients with PAMT.

Variable	Classification		Z values	*p* values
VAS score 6 mo*	Type IV	Type I	-1.964	0.201
		Type II	-2.157	0.135
		Type III	-0.161	0.999
	Type III	Type I	-2.267	0.106
		Type II	-2.479	0.063
	Type II	Type I	0.063	0.999
VAS score 12 mo*	Type IV	Type I	-2.763	**0.029**
		Type II	-2.708	**0.034**
		Type III	-0.571	0.940
	Type III	Type I	-2.768	**0.029**
		Type II	-2.679	**0.037**
	Type II	Type I	-0.427	0.974

Bold values means p< 0.05.

### 3.3 Surgical results

The operative time of 46 patients was 110min (90.0, 130.0), blood loss was 85ml (60.0, 112.5 ml), and the PHS was 6 days (5, 7 days). As for surgical data, the Kruskal–Wallis non-parametric tests suggested that there was a significant difference in means of operation time (H =9.607, *p* =0.022), EBL (H =12.386, *p* =0.006), and PHS (H =10.581, *p* =0.014) about different types of tumors (**Table 2**). According to DSCF tests, type IV was less than type III in EBL (Z =-3.041, *p* =0.013) and PHS (Z =-3.003, *p* =0.014); and type IV was also less than type II about operation time (Z =-2.653, *p* =0.040) (**Table 4**). Postoperative complications were cerebrospinal fluid leakage in 2 cases, cavitary effusion in the operative area in 5 cases, and incisional infection in 3 cases, which were relieved with physiotherapy and medication (**Table 2**).

**Table 4:** Influence of different types on operation of patients.

Variable	Classification		Z values	*p* values
Operation time (min)	Type IV	Type I	-1.450	0.468
		Type II	-2.653	**0.040**
		Type III	-2.405	0.076
	Type III	Type I	0.571	0.940
		Type II	-0.586	0.936
	Type II	Type I	0.876	0.817
EBL (ml)	Type IV	Type I	-0.575	0.940
		Type II	-2.535	0.055
		Type III	-3.041	**0.013**
	Type III	Type I	1.772	0.287
		Type II	0.845	0.833
	Type II	Type I	1.226	0.610
PHS (days)	Type IV	Type I	-1.743	0.301
		Type II	-1.706	0.320
		Type III	-3.003	**0.014**
	Type III	Type I	0.108	0.999
		Type II	-1.478	0.451
	Type II	Type I	1.784	0.281

Bold values means p< 0.05.

## 4 Discussion

Traditional open surgery is the main choice for lumbar dumbbell tumors, where total tumor resection and instrument fixation is accomplished by excising the facet joints and opening the intervertebral foramen (5). For lumbar dumbbell-shaped tumors, most surgeons currently believe that minimally invasive spinal surgery (MISS) can achieve the same therapeutic effect as open surgery (6–8). Among them, the paravertebral approach and micro-tubular technique is promising for the treatment of lumbar dumbbell tumors.

Tubular retractors have different diameters sizes (14-28 mm) and are also divided into non-expandable and expandable types. The choice of tubule during surgery is based on the size, location, and pathological type of the lesion. Tubular spine surgery is performed using a tubular retractor to reach the lesion and provide sufficient space for manipulation to remove the lesion while reducing normal tissue damage without increasing the risk of nerve injury.

Zairi et al. applied a non-expandable tubule and an expandable tubule (24 mm in diameter) respectively to complete 8 cases of lumbar dumbbell-shaped tumor resection with an average maximum tumor diameter of 3-4 cm (9). Lee et al. completed GTR of subdural extramedullary schwannomas using a tubular retractor (6 cases) or unilateral hemilaminectomy (18 cases) (10). We have previously performed GTR of 56 subdural extramedullary schwannomas under the tubule (11) and also performed 31 resections of thoracic dumbbell-shaped tumors (Eden II-IV) under the micro-tubule (12). Thavara et al. reported 13 subdural tumor tubule (diameter: 22 mm-30 mm) surgeries, of which 8 (67%) completed GTR and 4 cases (33%) completed NTR, mainly because a small section of tumor tissue adhered tightly to the spinal cord and nerve roots (13). In Eden II group, the intradural nerve root of three cases was invaded by the tumor and could not be dissected; an intraoperative electrophysiological monitoring stimulator was applied to confirm that invaded nerve root by the tumor was a sensory nerve root. Then, the sensory nerve root was resected along with tumors, and there was no new symptom after surgery.

By adjusting the position and angle of the paravertebral intramuscular pathway to achieve resection of intraspinal, foraminal and paravertebral tumor tissue, microtubular surgery was performed in 46 (100%) cases of total resection of lumbar dumbbell-shaped tumors (Eden I-IV types) in one stage in Eden type II and III tumors, the establishment of 2 paravertebral muscle tubular paths (dual-tubule path) was the key to complete lumbar intraspinal and paravertebral tumor resection, and it can avoid damage to the joints facet and isthmus, avoid excessive stretch and separation of muscles, reduce muscle damage, and reduce the risk factors for spinal instability (14, 15). However, we still recommend combined surgery or mini-open surgery for tumors that may invade the spinal cord, have large tumor size, or have abundant blood supply (5, 16).

Among the lumbar spine dumbbell-shaped tumors of different Eden classifications, PAMT surgery was slightly different in terms of operative time, EBL and PHS. We found that the operative time, EBL, and PHS of Eden II were higher than those of Eden III or Eden IV. The main reasons were more time required for resecting the intradural tumor, suturing of the dura, more bleeding from the epidural plexus, and longer bed rest after surgery, respectively, in Eden II. Since the tumor in Eden IV was located in the paravertebral region, the operation did not need to deal with intraspinal lesions, the intravertebral venous plexus was not affected, and operative time and EBL were the lowest, and postoperative recovery was also the fastest. When comparing the improvement in VAS scores 12 months after PAMT, types I and II showed more improvement than types III and IV, respectively, probably because the preoperative tumor portion of the tumor located in the spinal canal compressed and irritated the nerve roots more severely in types I and II, leading to a higher chance of back or leg pain symptoms, with greater severity. For types III and IV, some patients did not have back and leg pain before surgery, so the VAS score improved less after surgery.

During the surgery on 2 schwannomas (Eden IV), we exposed the outer border of the tumor capsule and then separated the tumor along with the tumor capsule and resected the tumor in pieces, and found more bleeding from the tumor capsule and tumor tissues compared with previous surgeries. Subsequently, in three other schwannomas (Eden IV), we intentionally separated and resected the tumor along with the medial border (near the spinal canal) of tumor capsule and found that intraoperative bleeding could be significantly reduced. Therefore, we performed the intracapsular resection sequence for paravertebral tumors by first separating and resecting the tumor from the medial border (near the spinal canal) of tumor, separating towards the cranial and caudal ends, respectively, and finally reaching the lateral border of the capsule. This sequence of resection operations reduced intraoperative bleeding during the subsequent resection of paravertebral tumor tissue for lumbar dumbbell-shaped tumors (Eden II-IV). We considered that it may be that the vascular network formed by the paravertebral arteries and veins was compressed and pushed out of place by the tumor, and that removal of the tumor starting from the lateral border of the tumor reduced the compression of the vascular network, making it easier to bleed distally and that the tumor blood supplying vessels and draining veins were mainly located on the paravertebral side and more often anastomose and communicating with the normal vascular network on the paravertebral side. Therefore, intracapsular resection of the tumor from the paravertebral side may reduce intraoperative bleeding by blocking the tumor blood supplying vessels and draining veins earlier, and reducing bleeding in the normal paravertebral vascular network by compression, but there was no evidence to confirm this conclusion.

The main methods of neurophysiological monitoring in spinal surgery are EMG, SSEP, and MEP. Intraoperative evoked potential monitoring can provide important warning signals early, but each electrophysiological monitoring technology has its own limitations, such as the high false-negative rate of SSEP, especially in patients with preoperative nerve damage, the reliability of SSEP is significantly reduced (17, 18). Our combined intraoperative continuous monitoring of EMG, SSEP, and TcMEP can determine whether the sensory and motor conduction pathways are damaged, so as to adjust or suspend the surgical operation and take protective measures to prevent postoperative neurological dysfunction. We performed INMO on 317 intravertebral lesions and found that intraoperative monitoring improved in 22 cervical patients and 5 thoracic patients, and only one case of lumbar giant neurogenic tumor had improved intraoperative monitoring (19). In 46 lumbar dumbbell-shaped tumor surgeries, there was no significant difference in the postoperative JOA scores and VAS scores between the 20 patients with combined intraoperative neurophysiological monitoring and those without intraoperative electrophysiological monitoring, probably because the tumors were small in size, and it did not invade the conus medullaris, most of the tumors originated from sensory roots, and the tumor-bearing nerve functions overlapped and crossed with the functions of the nerve roots of the adjacent segments.

As with other spinal surgical methods, microtubular spine surgery carried the risk of perioperative complications, including tumor residual or recurrence, hemorrhage, neurological damage, cerebrospinal fluid leakage, thrombosis, infection, and instability. Among these complications, cerebrospinal fluid leakage was a more serious surgical complication that may cause mental disorders, headaches, dizziness, and central system infection in patients (20–22).

Thavara et al. reported 1 case (7.7%, 13 cases) of cerebrospinal fluid leakage and pseudomeningocele during tubular surgery, which resolved after 10 days of conservative treatment and 1 case of wound infection (7.7%, 13 cases) that healed after re-suturing (13). Zairi et al. reported 1 case (12.5%,8 cases) of intraoperative cerebrospinal fluid leakage that resolved after fat tissue packing and glue reinforcement (9). Xu et al. had 2 cases (11.8%, 17 cases) of cerebrospinal fluid leakage during expandable tubular surgery for thoracic meningiomas, which were cured after conservative treatment including drainage (23). Of the 46 cases in this group, 2 cases (4.3%) had postoperative cerebrospinal fluid leakage, mainly due to inadequate suture of dural incision, diabetes, and muscle atrophy and weakness, and the patients were cured after 7-9 days of conservative treatment.

Our experience in reducing cerebrospinal fluid leakage after surgery was as follows (1): 6-0 nylon thread was used to suture the dura intermittently in the dural incision, and the incision was reinforced with epidural bioprotein glue and a meningeal patch (2); using the muscle splitting technique to separate the paravertebral muscles to reduce muscle damage, which helps to reset the muscles after exiting the tubule, and to close the surgical space and surgical path (3); The muscle fascia and skin are tightly sutured (4); The drainage tube does not go through the tubular path, and it was removed as early as possible within 2 days. 5 cases (10.9%) in this group were found to have cavity effusion in the operation area by MRI re-examination, considering the large tumor volume and slow muscle retraction after resection, the effusion caused by the residual cavity disappeared after 6 months of follow-up. In addition, in tubular spine surgery the significantly reduced tissue damage and the layer-by-layer suturing of muscle, subcutaneous, and skin could reduce the cavity in the surgical area, which was helpful for healing of postoperative cerebrospinal fluid leakage and effusion in the surgical area (24).

### 4.1 Limitations

The PAMT was not suitable for all lumbar spine dumbbell-shaped tumor surgeries. For example, large paravertebral tumors (>5 cm in diameter), tumors invading multiple intervertebral foramina or vertebral segments, tumors with abundant blood supply, and scoliosis deformity made tubular spine surgery riskier than that of open surgery and difficult for one-stage excision. These types of tumors require combined surgery or mini-open surgery to completely remove the tumor, and if necessary, fusion and instrument fixation surgery. In addition, the cases in the study involved different pathological types. Nevertheless, in surgery for lumbar dumbbell-shaped tumors with different pathological types, we found that the pathological type had little influence on the choice of surgical approach when choosing a minimally invasive tubular spinal option, but the purpose of surgery was slightly different for different pathological types. The majority of lumbar dumbbell-shaped tumors are benign tumors, whether nerve sheath tumors, neurofibromas or ganglioneuroblastomas where the surgical objectives are total removal of the tumor and preservation of spinal stability. In cyst lesions, the prevention of cerebrospinal fluid leakage is one of the keys to surgery. In metastatic tumors, the goal of surgery is to obtain pathology and neurological decompression, which the patient got the time to undergo subsequent radiotherapy or Chemotherapy treatment. Fortunately, none of the metastatic tumors in our group have experienced tumor recurrence *in situ* during follow-up. If the tumor tissue is rich in blood supply and the view under the tubule is unclear and prone to medically induced injury, access spine surgery is not recommended and is instead replaced by conventional surgery.

The number of cases collected in this group was small, and some cases were followed up for less than 2 years. In the future, a larger number of cases and a longer follow-up are needed to verify the advantages and disadvantages of tubular spine surgery. We did not find an improvement in patient prognosis with the application of combined intraoperative neurophysiological monitoring techniques, which may be related to the small number of cases and the lack of randomization. However, we found that the application of continuous intraoperative neurophysiological monitoring increased the operative time but provided a powerful aid to the surgeon for judging nerve function and whether to remove the tumor-bearing nerve.

## 5 Conclusions

This study is the first to comprehensively validate PAMT for different types of small lumbar dumbbell-shaped tumors, including Eden types I-VI. and PAMT can achieve complete resection of the tumor in one stage and a good prognosis. In addition, PAMT does not require additional internal fixation, and no lumbar instability was found during follow-up. The promotion of this technology is expected to reduce the financial burden on patients.

## Data availability statement

The raw data supporting the conclusions of this article will be made available by the authors, without undue reservation.

## Ethics statement

Approval for this study was granted by the Ethics committee of Fujian Medical University Union Hospital, Fuzhou, China (2020YF023-01). The patients/participants provided their written informed consent to participate in this study.

## Author contributions

CC and RW developed the concept of the manuscript. ZL and RW conducted the analysis of the data, then RW, ZL, XX, YC composed and edited the manuscript text, figures, and tables. All authors contributed to the article and approved the submitted version.

## Funding

This work was funded by Technology and Innovation Foundation of Fujian, China (grant no 2021Y0021 to RW).

## Conflict of interest

The authors declare that the research was conducted in the absence of any commercial or financial relationships that could be construed as a potential conflict of interest.

The reviewer KL declared a shared parent affiliation with the authors to the handling editor at the time of review.

## Publisher’s note

All claims expressed in this article are solely those of the authors and do not necessarily represent those of their affiliated organizations, or those of the publisher, the editors and the reviewers. Any product that may be evaluated in this article, or claim that may be made by its manufacturer, is not guaranteed or endorsed by the publisher.

## References

[1] Wang R Liang ZY Chen Y Chen CM. Comparison of the Clinical Efficacy of Transforaminal Endoscopy and Microtubular Technology for the Treatment of Lumbar Dumbbell-Shaped Tumors. Neurospine (2022) 19(3):513–23. doi: 10.14245/ns.2244152.076 PMC9537841 35577331

[2] Nanda A Kukreja S Ambekar S Bollam P Sin AH. Surgical strategies in the management of spinal nerve sheath tumors. World Neurosurg (2015) 83:886–99. doi: 10.1016/j.wneu.2015.01.020 25655687

[3] Ghadirpour R Nasi D Iaccarino C Romano A Motti L Sabadini R. Intraoperative neurophysiological monitoring for intradural extramedullary spinal tumors: predictive value and relevance of d-wave amplitude on surgical outcome during a 10-year experience. J Neurosurg Spine (2018) 30(2):259–67. doi: 10.3171/2018.7.SPINE18278 30497134

[4] Kang H. Sample size determination and power analysis using the G*Power software. J Educ Eval Health Prof. (2021) 18:17. doi: 10.3352/jeehp.2021.18.17 34325496 PMC8441096

[5] Safaee M Oh T Barbaro NM Chou D Mummaneni PV Weinstein PR. Results of spinal fusion after spinal nerve sheath tumor resection. World Neurosurg (2016) 90:6–13. doi: 10.1016/j.wneu.2016.01.015 26802866

[6] Gandhi RH German JW. Minimally invasive approach for the treatment of intradural spinal pathology. Neurosurg Focus (2013) 35:E5. doi: 10.3171/2013.5.FOCUS13163 23905956

[7] Berenson J Pflugmacher R Jarzem P Zonder J Schechtman K Tillman JB. Balloon kyphoplasty versus non-surgical fracture management for treatment of painful vertebral body compression fractures in patients with cancer: a multicentre, randomised controlled trial. Lancet Oncol (2011) 12:225–35. doi: 10.1016/S1470-2045(11)70008-0 21333599

[8] Mendel E Bourekas E Gerszten P Golan JD. Percutaneous techniques in the treatment of spine tumors: what are the diagnostic and therapeutic indications and outcomes? Spine (Phila Pa 1976) (2009) 34:S93–100. doi: 10.1097/BRS.0b013e3181b77895 19829281

[9] Zairi F Troux C Sunna T Karnoub MA Boubez G Shedid D. Minimally invasive resection of large dumbbell tumors of the lumbar spine: Advantages and pitfalls. Clin Neurol Neurosurg (2018) 168:91–6. doi: 10.1016/j.clineuro.2018.03.005 29529487

[10] Lee SE Jahng TA Kim HJ. Different surgical approaches for spinal schwannoma: a single surgeon's experience with 49 consecutive cases. World Neurosurg (2015) 84:1894–902. doi: 10.1016/j.wneu.2015.08.027 26325210

[11] Zhuang Y Cai G Fu C Zhang W Zhao W Wang R. Novel combination of paraspinal keyhole surgery with a tubular retractor system leads to significant improvements in lumbar intraspinal extramedullary schwannomas. Oncol Lett (2017) 14:7873–9. doi: 10.3892/ol.2017.7203 PMC5755262 29344232

[12] Wang R Chen Y Liang Z Yang W Chen C. Efficacy of one-stage paravertebral approach using a micro-tubular technique in treating thoracic dumbbell tumors. Orthop Surg (2021) 13:1227–35. doi: 10.1111/os.12991 PMC8274168 33943013

[13] Thavara BD Kidangan GS Rajagopalawarrier B. Analysis of the surgical technique and outcome of the thoracic and lumbar intradural spinal tumor excision using minimally invasive tubular retractor system. Asian J Neurosurg (2019) 14:453–60. doi: 10.4103/ajns.AJNS_254_18 PMC6516036 31143261

[14] Ishikawa Y Ohashi M Hirano T Matsuda M Akabane T Kanno H. Mid- to long-term outcomes after resection of thoracic dumbbell tumors managed by laminectomy and unilateral total facetectomy without instrumented fusion. Global Spine J (2021) 11:21925682211008836. doi: 10.1177/21925682211008836 PMC10240595 33973481

[15] Vergara P. A novel less invasive technique for the excision of large intradural and extradural dumbbell lumbar schwannomas: the “dual approach”. World Neurosurg (2016) 95:171–6. doi: 10.1016/j.wneu.2016.07.103 27506400

[16] Moses ZB Barzilai O O'Toole JE. Benign intradural and paraspinal nerve sheath tumors: advanced surgical techniques. Neurosurg Clin N Am (2020) 31:221–9. doi: 10.1016/j.nec.2019.11.002 32147013

[17] Takahashi M Imagama S Kobayashi K Yamada K Yoshida G Yamamoto N. Validity of the alarm point in intraoperative neurophysiological monitoring of the spinal cord by the monitoring working group of the japanese society for spine surgery and related research: a prospective multicenter cohort study of 1934 cases. Spine (Phila Pa 1976) (2021) 46:E1069–76. doi: 10.1097/BRS.0000000000004065 34559750

[18] Lee S Cho DC Rhim SC Lee BJ Hong SH Koo YS. Intraoperative monitoring for cauda equina tumors: surgical outcomes and neurophysiological data accrued over 10 years. Neurospine (2021) 18:281–9. doi: 10.14245/ns.2040660.330 PMC8255760 34218610

[19] Wi SM Lee HJ Kang T Chang SY Kim SM Chang BS. Clinical significance of improved intraoperative neurophysiological monitoring signal during spine surgery: a retrospective study of a single-institution prospective cohort. Asian Spine J (2020) 14:79–87. doi: 10.31616/asj.2019.0025 31694354 PMC7010504

[20] Zuckerman SL Laufer I Sahgal A Yamada YJ Schmidt MH Chou D. When less is more: The indications for MIS techniques and separation surgery in metastatic spine disease. Spine (Phila Pa 1976) (2016) 41 Suppl 20:S246–53. doi: 10.1097/BRS.0000000000001824 PMC5551976 27753784

[21] Ottenhausen M Ntoulias G Bodhinayake I Ruppert FH Schreiber S Förschler A. Intradural spinal tumors in adults-update on management and outcome. Neurosurg Rev (2019) 42:371–88. doi: 10.1007/s10143-018-0957-x 29455369

[22] Soriano Sánchez JA Soto García ME Soriano Solís S Rodríguez García M Trejo Huerta P Sánchez Escandón O. Microsurgical resection of intraspinal benign tumors using non-expansile tubular access. World Neurosurg (2020) 133:e97–104. doi: 10.1016/j.wneu.2019.08.170 31505279

[23] Xu J Yu BF Liu CH Zheng W Xiao YH Lin Y. Microscopic keyhole technique for surgical removal of thoracic spinal meningiomas. World Neurosurg (2019) 3:S1878-8750(18)32922-X. doi: 10.1016/j.wneu.2018.12.099 30610972

[24] Müller SJ Burkhardt BW Oertel JM. Management of dural tears in endoscopic lumbar spinal surgery: a review of the literature. World Neurosurg (2018) 119:494–9. doi: 10.1016/j.wneu.2018.05.251 29902608

